# Integrated Additive Manufacturing of TGV Interconnects and High-Frequency Circuits via Bipolar-Controlled EHD Jetting

**DOI:** 10.3390/mi16080907

**Published:** 2025-08-02

**Authors:** Dongqiao Bai, Jin Huang, Hongxiao Gong, Jianjun Wang, Yunna Pu, Jiaying Zhang, Peng Sun, Zihan Zhu, Pan Li, Huagui Wang, Pengbing Zhao, Chaoyu Liang

**Affiliations:** 1 State Key Laboratory of Electromechanical Integrated Manufacturing of High-Performance Electronic Equipments, Xidian University, Xi’an 710071, China; baidq@stu.xidian.edu.cn (D.B.);; 2 School of Construction Machinery, Chang’an University, Xi’an 710064, China; liangcy@chd.edu.cn

**Keywords:** electrohydrodynamic (EHD) printing, through-glass via (TGV) filling, glass-integrated RF antenna

## Abstract

Electrohydrodynamic (EHD) printing offers mask-free, high-resolution deposition across a broad range of ink viscosities, yet combining void-free filling of high-aspect-ratio through-glass vias (TGVs) with ultrafine drop-on-demand (DOD) line printing on the same platform requires balancing conflicting requirements: for example, high field strengths to drive ink into deep and narrow vias; sufficiently high ink viscosity to prevent gravity-induced leakage; and stable meniscus dynamics to avoid satellite droplets and charge accumulation on the glass surface. By coupling electrostatic field analysis with transient level-set simulations, we establish a dimensionless regime map that delineates stable cone-jetting regime; these predictions are validated by high-speed imaging and surface profilometry. Operating within this window, the platform achieves complete, void-free filling of 200 µm × 1.52 mm TGVs and continuous 10 µm-wide traces in a single print pass. Demonstrating its capabilities, we fabricate transparent Ku-band substrate-integrated waveguide antennas on borosilicate glass: the printed vias and arc feed elements exhibit a reflection coefficient minimum of −18 dB at 14.2 GHz, a −10 dB bandwidth of 12.8–16.2 GHz, and an 8 dBi peak gain with 37° beam tilt, closely matching full-wave predictions. This physics-driven, all-in-one EHD approach provides a scalable route to high-performance, glass-integrated RF devices and transparent electronics.

## 1. Introduction

Electrohydrodynamic (EHD) jet printing exploits strong electric fields to pull a fine liquid jet from a nozzle, forming a stable Taylor cone that can deposit functional inks (1–10,000 cP) with sub-micron linewidths in a mask-free, drop-on-demand manner. The driving principle of EHD jet printing is the action of Maxwell stresses on a leaky-dielectric ink, as described by the Taylor–Melcher model. Above a critical electric stress, free charges accumulate at the liquid–air interface, deforming the meniscus into a conical shape (the Taylor cone) and emitting a fine jet when the normal Maxwell stress overcomes surface tension [1,2,3]. EHD jetting operates in two primary modes: in continuous-jet mode, a steady Taylor cone emits an uninterrupted liquid stream, whereas in drop-on-demand (DoD) mode, brief voltage pulses generate discrete droplets that, when synchronized with precise substrate motion, draw patterns or circuit lines only where and when needed [4,5]. This versatility has enabled applications ranging from high-resolution interconnects and sensor arrays on flexible substrates [6] to transparent metal-mesh electrodes for smart glasses and touch panels with >90% optical transmission and RF-grade conductivity [7]. Recent advances include scalable, parallelized multi-nozzle arrays that overcome throughput bottlenecks by mitigating field crosstalk [8], and transient plasma-impingement EHD that creates “virtual electrodes” for true 3D conformal printing on curved dielectrics at sub-500 nm resolution [9].

Transparent glass substrates combine exceptional optical transparency, low dielectric loss and high surface smoothness, making them an attractive platform for next-generation antenna and interconnect technologies [10]. In recent years, a variety of glass-based antenna concepts have been demonstrated: Hu et al. reported tri-band (2.4/5.2/5.8 GHz) glass dielectric resonator antennas with polarization, pattern and space diversity for WiFi MIMO applications [11]; Jang et al. developed an on-glass 5G monopole antenna directly printed onto vehicle window glass using a CPW feed and inductive line phasing to overcome the high dielectric loss of laminated automotive glass [12]; Su et al. achieved a compact 77 GHz automotive radar antenna by stacking five glass wafers interconnected with TGV and redistribution layers [13]; Morimoto et al. designed an optically transparent metal-mesh slot antenna compatible with lossy active lens stack-ups for smart glasses, demonstrating >−4.5 dB efficiency at 2.4 GHz [14]; and Chaloun et al. comprehensively reviewed the emergence of RF glass technologies—covering material systems, TGV formation, glass-interposer fabrication and millimeter-wave packaging/applications—highlighting both opportunities and remaining challenges for mainstream adoption [10]. EHD drop-on-demand printing has also been explored as a void-free metallization route. Hussain et al. demonstrated Ag nanoparticle deposition in TGVs with aspect ratios up to 16.3 by optimizing jetting voltage, curing temperature, and multi-step fill-evaporate cycles, yielding complete, void-free metallization and resistances as low as 20 mΩ for AR  =  6.25 [15]. Such additive approaches benefit from mask less patterning and reduced waste, but require careful control of electric Bond number, ink rheology and process throughput to avoid defects [16,17,18].

TGVs have emerged as a key enabling technology for three-dimensional (3D) integration in both microelectronics and emerging transparent devices, offering low dielectric loss, high thermal stability, and optical transparency compared to silicon-based interposers [19,20]. Conventionally, TGV metallization relies on seed-layer deposition (e.g., by PVD or electroless methods) followed by Cu electroplating; however, achieving void-free filling in high-aspect-ratio vias often requires complex additive chemistries, pulse-reverse plating strategies, and post-deposition chemical-mechanical planarization (CMP), which increases process cost and can introduce surface defects [15,21,22]. Li et al. demonstrated a mask-free EHD printing strategy—combining a PDMS bottom coat, oxygen-plasma pretreatment, and optimized 5 kV/15 s parameters—to metallize through-glass vias with void-free filling and <0.1 Ω resistance, but their study was limited to low depth-to-diameter ratios (≤3:1) [23].

Currently, EHD jet printing for void-free filling of TGVs and simultaneous micropatterning of fine lines faces several challenges. Driving metal-nanoparticle inks into high-aspect-ratio vias requires very strong electric fields, but excessive field strength induces cone-jet instabilities and satellite droplets. Conversely, preventing leakage or voids—caused by gravity or substrate surface-tension differences—necessitates high-viscosity inks, which in turn raise the jetting onset voltage and reduce printing frequency, limiting resolution. In addition, charge accumulation on the glass surface can deflect droplets and interrupt line continuity, degrading continuous-line quality. To date, most studies have been unable to balance ink rheology, nozzle geometry, and driving waveform, and have not achieved both complete TGV filling and sub-10 μm line printing in a single EHD process.

In this work, we couple numerical electrostatic field calculations with interface-tracking simulations to map the evolution of the liquid meniscus under varying voltage, ink viscosity, and nozzle geometry. By correlating these predictions with real-time imaging and post-print profilometry, we establish a unified process window that clearly defines the boundaries between no-jetting, pulsating meniscus behavior, stable cone-jet operation, and over-jetting regimes. This experimentally validated regime map serves as a powerful design tool for achieving defect-free, void-free filling of high-aspect-ratio vias and for reliably printing fine-pitch conductive lines.

Leveraging the insights from this combined modeling-experimental framework, we demonstrate direct EHD fabrication of transparent substrate-integrated waveguide antennas on glass. Operating within the identified process window, the platform produces uniform metallization of vertical interconnects alongside micron-scale circuit features in a single pass, without recourse to masking or multi-step lithography. Electromagnetic characterization of the printed antennas shows deep resonance notches and broad impedance bandwidths in the intended frequency band, confirming that our approach translates predictive jet-dynamics control into high-performance, glass-integrated RF devices.

## 2. Methods

### 2.1. EHD Filling and Printing Scheme

**Figure 1:**
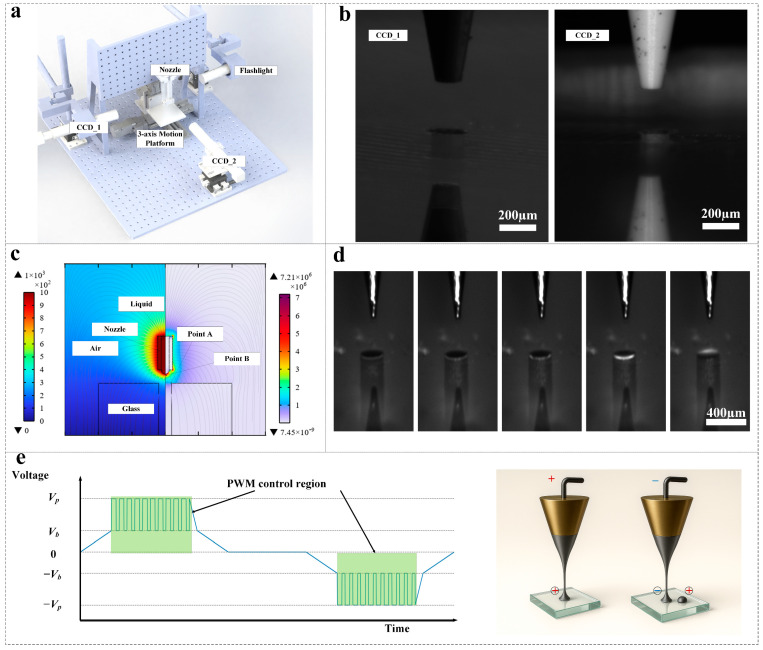
Schematic and results of EHD via filling and circuit printing. (**a**) Platform schematic. (**b**) The nozzle tip aligned with the glass via. (**c**) COMSOL 6.2 2D electrostatic field distribution. (**d**) Sequence of EHD jetting entering the TGV. (**e**) Drop-on-demand printing driven by PWM-combined bipolar voltage.

To realize both void-free TGV metallization and simultaneous glass-integrated waveguide patterning, we first describe the core electrohydrodynamic (EHD) printing scheme, detailing its fundamental principles and key parameter choices. In Figure 1a, we present the fully integrated EHD printing platform, engineered for both high-precision via filling and fine-pitch circuit patterning on glass substrates. At the heart of the setup lies a vibration-damped granite optical breadboard, upon which a closed-loop, three-axis linear stage (X–Y–Z travel range: 200 × 200 × 30 mm; positioning repeatability ≤ 0.5 μm) provides submicron control of the substrate.

The system employs two distinct ink-supply schemes on a single carriage, allowing seamless switching between high-viscosity filling and low-viscosity writing without realignment. High-viscosity silver-nanoparticle suspensions (500–8000 cP) are delivered by a precision flow pump coupled to a stepper-motor–actuated pressure regulator, yielding stable Taylor-cone formation and reproducible flow rates up to 200 nL/s. Low-viscosity inks (10–500 cP) are fed from a gravity-driven U-tube reservoir.

Real-time monitoring and feedback are provided by two orthogonally oriented, high-speed CMOS cameras. CCD_1, aligned coaxially with the nozzle axis, captures jet dynamics at up to 10,000 fps for process diagnostics and post-processing analysis. To guarantee sub-micrometer registration between the EHD nozzle and each TGV, we employ two high-resolution CMOS cameras mounted at 90° to one another. CCD_2 (side view) is positioned laterally to capture the elevation and vertical centering of the nozzle tip relative to the via entrance. CCD_1 (coaxial view) is aligned along the nozzle axis to monitor lateral (X–Y) positioning and meniscus contact with the via rim, with a 2 µm/pixel resolution over a 1 × 1 mm window, as Figure 1b shows.

Prior to each print cycle, the system triggers both cameras and a synchronized strobe flash (exposure 5 µs) to capture instantaneous, distortion-free snapshots of the nozzle-substrate interface. Custom image-processing algorithms then extract the nozzle tip coordinates and compare them to pre-calibrated TGV centroids (determined via a 50 µm-pitch calibration grid). By iteratively adjusting the three-axis stage in closed-loop feedback—using sub-pixel interpolation—the platform achieves alignment accuracy better than ±2 µm in all directions. This dual-view strategy ensures that the Taylor cone consistently forms at the center of the via, eliminating off-center deposition and preventing voids or bridging defects during the high-viscosity ink fill.

In the COMSOL model (Figure 1c), We couple steady-state electrostatics with transient two-phase flow using the level-set method, an interface capturing approach that implicitly represents the fluid–air boundary via a signed-distance function and naturally handles topological changes. This framework simultaneously resolves the electric field distribution and the meniscus evolution during TGV filling. The governing equations are [24,25]:

$$\nabla \cdot \left(\varepsilon_{r}\varepsilon_{0}\nabla V\right)=0$$

where *V* is the electric potential, *ε*_0_ the vacuum permittivity and *ε_r_* the domain-dependent relative permittivity. The stainless-steel nozzle is treated as an equipotential conductor at high voltage, while all glass surfaces are grounded.

Next, fluid motion is governed by the incompressible Navier–Stokes and continuity equations augmented by Coulomb and surface-tension forces:

$$\rho \left(\frac{\partial u}{\partial t}+u\cdot \nabla u\right)=-\nabla p+\nabla \cdot \left[\mu \left(\nabla u+\nabla u^{T}\right)\right]+\rho_{e}E+\sigma \kappa \delta (\phi )n,\;\nabla \cdot u=0$$

where **u** is velocity, *p* pressure, *ρ* density, *μ* dynamic viscosity, $\rho_{e}=\nabla \cdot \left(\varepsilon_{r}\varepsilon_{0}E\right)$ the local charge density, E=−∇V the electric field, *σ* the interfacial tension, *κ* the interface curvature, *δ(ϕ)* a Dirac delta function localizing the surface force, and n=∇ϕ/|∇ϕ| the unit normal at the interface.

Interface tracking employs the level-set transport equation:

$$\frac{\partial \phi }{\partial t}+u\cdot \nabla \phi =\gamma \nabla \cdot (\varepsilon \nabla \phi )$$

with reinitialization to enforce |∇ϕ|=1. Here, *ϕ* = 0 denotes ink, *ϕ* = 1 air, *γ* is a stabilization coefficient, and *ε* controls the diffuse-interface thickness.

Simulations proceed in two stages: a stationary study to compute the electric field, followed by a transient two-phase flow simulation over a few milliseconds (with microsecond-scale time steps) to capture jet initiation, cone formation and complete via filling. Field magnitude and interface shape are recorded at the Taylor-cone apex and via corner for direct comparison with high-speed imaging.

We employ two dimensionless parameters to unify our jetting data across different inks and operating conditions. The first is the dimensionless flow rate [26]:

$$Q^{*}=Q/Q_{0}$$

where *Q* is the volumetric ink throughput and *Q*_0_ is a characteristic flow scale set by the fluid density *ρ*, vacuum permittivity and electrical conductivity of the ink *K*:

$$Q_{0}=\frac{\sigma \varepsilon_{0}}{\rho K}$$

The second is the electric Bond number:

$$Bo_{E}=\varepsilon_{0}E^{2}D_{0}/(2\sigma )$$

which measures the ratio of Maxwell stress in the liquid meniscus to capillary pressure, with *E* denoting the local electric field strength at the nozzle tip, *D*_0_ the nozzle diameter, and *σ* the ink’s surface tension.

Figure 1d presents representative, time-resolved images of the EHD-driven filling sequence, as recorded by CCD_1 under synchronized strobe illumination. At the outset, a stable Taylor cone forms at the nozzle tip; as the applied field increases, a focused jet issues towards the TGV entrance. Subsequent frames capture droplet coalescence at the via mouth and the advancing ink meniscus, driven by both electric stresses and capillary forces, until the via is completely filled and the surface leveled. This dynamic sequence reveals the kinetics of jet impingement, wetting, and capillary infiltration. Ex situ cross-sectional SEM and nano-CT characterization subsequently confirm that the filling is conformal and free of voids, validating the high-fidelity of the in situ imaging observations.

To achieve both fine droplet control and suppression of charge-induced defects, we combine pulse-width modulation (PWM) of the peak voltage *V*_*p*_ with a bipolar (alternating polarity) waveform. As shown on the left side of Figure 1e, the PWM stage chops the high-level plateau at +*V*_*p*_ into short “on” pulses and “off” intervals around a base bias *V*_*b*_. By adjusting the duty cycle (ratio of “on” time to total period), the effective amplitude—and thus the Taylor-cone jet–volume per pulse—can be finely tuned to control droplet size. On the right side of Figure 1e, successive pulses invert their polarity between +*V*_*p*_ and −*V*_*p*_. This bipolar alternation rapidly neutralizes residual surface charge on the substrate between pulses, preventing the buildup of charge clouds that otherwise repel incoming droplets or destabilize the cone–jet, and thereby greatly improving both spatial accuracy and line continuity in printing.

Having established the core principles and critical parameters of the EHD on-demand printing method, we now outline how these are implemented in the overall fabrication workflow.

### 2.2. Process Workflow

**Figure 2:**
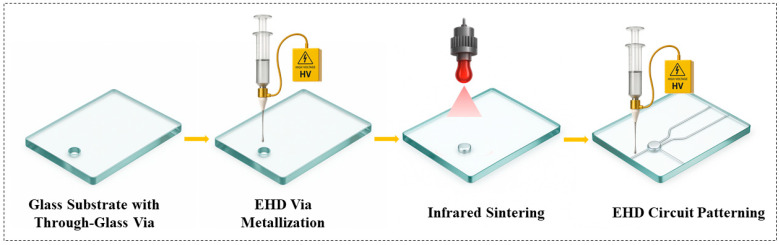
Process flow of EHD filling and printing.

Building on the method described above, this section details the overall process workflow—from substrate preparation through infrared sintering—that implements the EHD parameters to achieve void-free TGV filling and fine-pitch circuit writing. The overall process flow is shown in Figure 2. The process begins with a borosilicate glass substrate into which TGVs are formed by precision laser ablation. Next, under a high-voltage field, a high-viscosity silver-nanoparticle ink is EHD jetted into each via, producing a stable Taylor cone and achieving void-free, uniform filling. The filled vias are then rapidly densified by infrared sintering, which coalesces the nanoparticles into a continuous conductive plug and removes organic binders. Finally, the same platform switches to EHD printing of a low-viscosity ink to write micron-scale traces on both sides of the glass in a single, mask-free operation, seamlessly integrating via metallization and fine-pitch circuit patterning. The parameters of the simulation model, test instruments and ink properties used in this study are presented in Supporting File S1.

### 2.3. SIW Antenna Design Methodology

Building on the high-resolution deposition capabilities of our EHD scheme, we can not only metallize through-glass vias but also directly print the conductive boundaries that define substrate-integrated waveguides. By precisely controlling nozzle motion, voltage waveform, and ink rheology (as detailed in Section 2.1), the same platform creates the SIW sidewalls and interconnect traces with the dimensional accuracy and conductivity required for RF operation. We therefore describe how these printed features are translated into a full antenna design: outlining the critical geometries, simulation framework, and performance criteria for our glass-based SIW antennas.

All full-wave electromagnetic simulations were carried out in Ansys HFSS (High-Frequency Structure Simulator) to predict both near-field and far-field behavior of our EHD-printed, TGV-filled antenna. The 3D model—including the glass substrate, filled via geometry and surface metallization—was constructed directly in HFSS, with material properties assigned as follows: glass (ε_r_ = ~3.8, tan δ = 0.0012), and sintered silver (σ = 3.1 × 10^6^ S/m).

The structure was enclosed within an air box extending at least λ_0/4 from all faces, and radiation boundaries were applied on each outer face. Excitation was provided via a 50 Ω wave port at the feed coaxial interface. An adaptive tetrahedral mesh was used, targeting a maximum element length of λ_min_/10 at the highest sweep frequency to ensure convergence (∆S_11_ < 0.02 dB between passes). A discrete frequency sweep from 12.5 to 16.5 GHz (101 points) was performed to extract S-parameters, surface current distributions, and far-field patterns. Post-processing of the simulated S-parameters and field plots allowed evaluation of impedance bandwidth, radiation efficiency, and the impact of via geometry on antenna performance.

**Figure 3:**
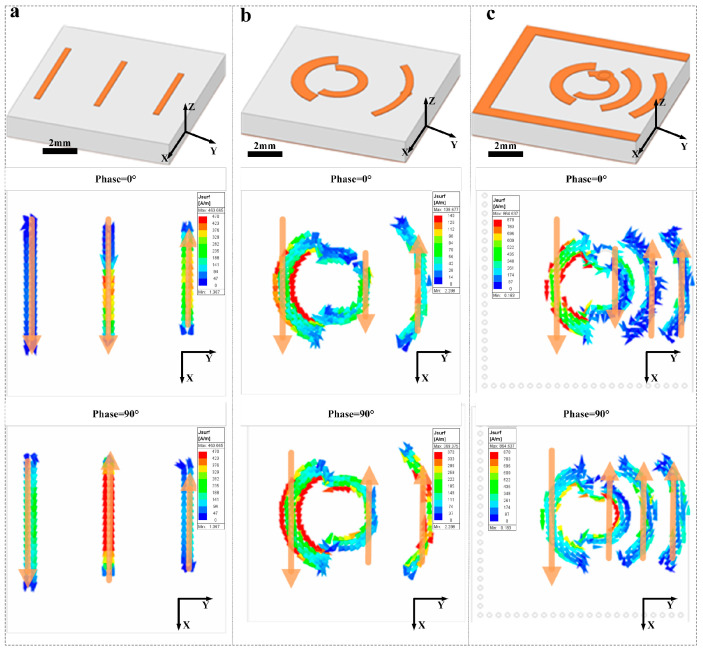
Evolution of radiator geometry and surface-current distributions. (**a**) Straight-element Yagi array. (**b**) Arc-shaped radiator (**c**) Semi-enclosed SIW cavity antenna.

Figure 3 shows how our radiator evolved in three stages on a 1.524 mm-thick substrate. We began with a conventional straight-element Yagi array (Figure 3a) [27], where 0°-phase currents cluster at the ends of each strip and rotate into the ±y plane at 90°, producing a classic end-fire beam along +x. To introduce mechanical flexibility without altering the beam pattern, we then bent each driver, reflector, and director into smooth circular arcs (Figure 3b). At both 0° and 90° phase states, the curved elements faithfully reproduce the same radiation direction and phase progression, demonstrating that the end-fire behavior is preserved on a bent geometry. Finally, we enclosed those arcs within a semi-closed SIW cavity (Figure 3c). Although the cavity walls carry negligible surface current, they serve as a passive reflector that enhances impedance matching, boosts gain, and shifts the beam further toward +y—yet the orthogonal-phase plots confirm that the original excitation phases and low cross-polarization are maintained throughout.

## 3. Results and Discussion

### 3.1. TGV Filling Performance Optimization

**Figure 4:**
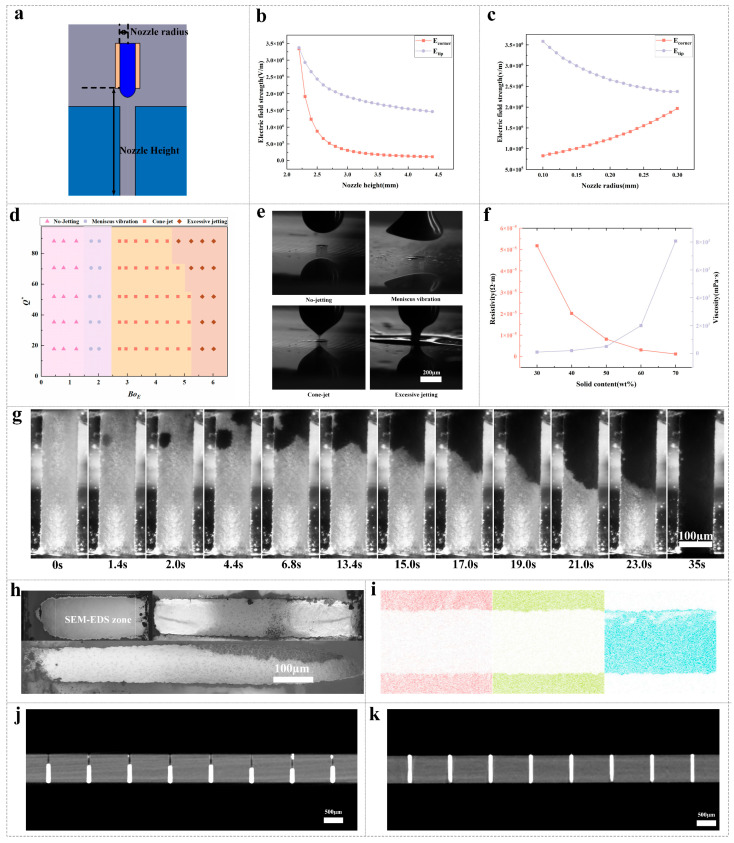
Influence of nozzle geometry, jetting regimes, ink properties, and via filling. (**a**) Schematic defining nozzle radius and nozzle height above the glass via. (**b**) Simulated versus nozzle height with nozzle radius 0.2 mm. (**c**) Simulated versus nozzle radius with nozzle height 2.4 mm. (**d**) Operational map showing four jetting regimes (no-jetting, meniscus vibration, cone-jet, excessive jetting) in parameter space. (**e**) Representative images of no-jetting, meniscus vibration, cone-jet, and excessive jetting. (**f**) Ink resistivity and viscosity plotted against solid content, with inset images of nano-CT at two concentrations. (**g**) Time-lapse sequence of EHD filling inside a via from 0 s to 35 s. (**h**) SEM views of fully filled vias. (**i**) Color-coded map of nanoparticle distribution. (**j**) Scan of an incompletely Filled TGV by Nano-CT. (**k**) Nano-CT scan of a completely filled TGV).

To elucidate the role of nozzle geometry on field localization, in Figure 4a, we define two key parameters: the nozzle height H, which is the vertical gap between the conductive nozzle tip—held at high voltage—and the electrically grounded bottom glass surface; and the nozzle inner radius R. Glass thickness is 2 mm. Steady-state electrostatic simulations then yield the field magnitudes at the Taylor-cone apex (*E*_tip_) and at the TGV corner (*E*_corner_). As shown in Figure 4b, both *E*_tip_ and *E*_corner_ decrease monotonically with increasing *H*. When *H* is below approximately 2.6 mm, the field strengths increase sharply—bringing the nozzle closer strongly concentrates the electric field at both the cone apex and via entrance. Beyond 2.6 mm, the decline becomes more gradual as the substrate’s influence on the field weakens. Importantly, heights smaller than ~2.2 mm, while producing very high apex fields, risk dielectric breakdown and excessive field gradients; heights above ~3.0 mm reduce *E*_tip_ below the threshold for stable cone-jet formation. We therefore identify an optimal height window of 2.2–2.8 mm, which maintains ≥80% of the maximum apex field while delivering ≥60% of the peak via field—ensuring both robust jet initiation and reliable via infiltration. Figure 4c shows the complementary effect of varying *R* at fixed height. Increasing *R* lowers the curvature-enhanced field at the apex (*E*_tip_)—due to a blunter emitter—but raises *E*_corner_ by spreading the equipotential boundary and directing more field lines toward the via. Very small radii (≲100 µm) over-concentrate the field, causing unstable, pulsatile jets; very large radii (≳250 µm) dilute the apex field below the jet-formation threshold. A mid-range radius of 120–180 µm strikes the best compromise, yielding controlled apex fields (~2.5 × 10^6^ V/m) and sufficient via corner fields (~1.0 × 10^6^ V/m).

Together, these results highlight a clear “sweet spot” for nozzle geometry: positioning the nozzle at ~2.4 mm above the glass with an inner radius of ~150 µm produces a stable Taylor cone, and minimizes the risks of satellite droplet formation or incomplete infiltration.

As shown in Figure 4d, we map our combined experimental and simulation data onto the dimensionless flow-rate (Q*) versus electric Bond number (*Bo*_*E*_) plane to delineate the distinct jetting regimes. At low Q* and low *Bo*_*E*_, the Maxwell stresses are insufficient to overcome surface tension, so the meniscus merely bulges without ejecting a jet (“no-jetting”). Increasing *Bo*_*E*_ at still-low flow rates produces periodic interface oscillations, but no sustained cone forms (“meniscus vibration”). Only within a narrow band of intermediate Q* and *Bo*_*E*_ does a stable Taylor cone emerge, issuing a continuous, well-focused jet ideal for rapid via filling (“stable cone-jet”). Beyond this window—at either excessive flow or overly strong fields—the jet destabilizes into satellite droplets, causing splatter and potential defects (“excessive jetting”).

Figure 4e presents representative high-speed CCD_1 frame under synchronized strobe illumination, each corresponding to one of these regimes. In the “no-jetting” snapshot, the ink interface deforms and retracts without emitting droplets; in “meniscus vibration,” the interface pulses but never acquires the pointed geometry of a Taylor cone. The “stable cone-jet” image clearly shows a sharp cone apex and a thin, steady jet, while the “excessive jetting” frame captures fragmented droplets detaching from an over-elongated jet. Together, these observations confirm that only the intermediate Q*–*Bo*_*E*_ window yields uniform, defect-free jets suitable for void-free TGV metallization, validating the regime map as a predictive tool for process parameter selection. To operate within the stable cone-jet window defined in the Q*–*Bo*_*E*_ map (Figure 4d), the applied voltage must be chosen so that the resulting field strength and flow rate combination falls squarely in that intermediate band. Since *Bo*_*E*_ ∝ *E*^2^ and *E* ∝ V one can back out the required voltage from the desired electric Bond number.

Figure 4f plots nano-silver ink resistivity (left axis) and viscosity (right axis) versus solid content (30–70 wt%). As the solid loading increases, resistivity drops steeply from about 5 × 10^−5^ Ω m at 30 wt% to 0.2 × 10^−5^ Ω m at 70 wt%, reflecting the formation of a more complete conductive network. To ensure void-free, gravity-resistant filling while maintaining excellent conductivity, we therefore select high-viscosity inks (≥2000 cP), corresponding to solid contents of 60 wt% or higher, for TGV filling.

Figure 4g presents a sequence of CCD_1 snapshots documenting the complete via-filling process using a 2000 cP silver-ink formulation under an applied voltage of 3.2 kV. At t = 0 s, the Taylor cone issues a well-focused jet that first wets the via entrance, forming a concave meniscus. Between 0 s and 2 s, the fill front advances rapidly, covering roughly 30–40% of the via depth as electric stress and capillary forces dominate viscous resistance. Thereafter, as seen at t = 4.4 s and 6.8 s, the front propagation rate decreases markedly: the increasing viscous drag within the narrowing annular gap and the rising capillary pressure across the diminishing curvature slow infiltration. The video of the filling process can be found in the Supporting files S2 and S3.

By 6.7 s, the meniscus has traversed approximately 70% of the via height, and further progress between 15.0 s and 19.0 s occurs in a creeping flow regime characterized by a nearly flat interface and slight sidewall pinning. At 21.0 s and 23 s, the fill front skirts the final 10% of the via, indicating that the combined Maxwell and capillary stresses are just sufficient to overcome the residual viscous barrier. Complete filling is reached by 35 s, with the final frame showing a smooth, level surface and no evidence of satellite-driven overspray.

These time-resolved observations underscore the trade-off inherent to high-viscosity inks: they support stable, void-free filling with minimal droplet breakup, but require prolonged field application to drive the late-stage meniscus. In practical terms, viscosities around 1500–2000 cP delineate the upper limit for rapid via filling (<35 s); beyond this, the infiltration time scales become prohibitive for high-throughput manufacturing.

SEM cross-sections of three through-glass vias with depth-to-diameter ratios of 2.8, 5.3 and 8.6 (left to right in the top row; the bottom panel shows a full-length view of the 8.6:1via) as shown in Figure 4h. Prior to imaging, all printed vias were subjected to infrared sintering at 250 °C for 10 min. In each micrograph the bright white region is the silver fill, dark gray is the glass wall. Even at the highest aspect ratio (8.6:1), the silver phase conforms tightly to the via walls, with only isolated sub-micron pores visible near the sidewalls. This uniform filling across all three geometries demonstrates that our optimized EHD parameters reliably overcome the increasing viscous and capillary resistances found in high-aspect-ratio channels.

Figure 4i shows the corresponding SEM-EDS maps for oxygen (red), silicon (green) and silver (blue) in the same vias. The red and green signals outline the glass sidewalls without penetrating the lumen, while the blue silver signal completely occupies the via interior in every case. The absence of oxygen or silicon within the blue regions confirms that no glass debris is entrained during filling and that the silver plug is compositionally pure. Together, these data validate that our 60 wt% silver formulation and operating conditions achieve void-free, uniform metallization even in vias with aspect ratios up to 8.6:1.

Analysis of the nano-CT cross-sections of sintered silver reveals a marked improvement in via metallization after process optimization. In the pre-optimization sample (Figure 4j), only about 50–60% of the via volume is occupied by sintered silver—significant voids and discontinuities remain—whereas in the optimized sample (Figure 4k) the filling fraction rises to 99%, with a fully continuous silver column from top to bottom and no detectable pores. Here, the filling fraction was quantified by segmenting the bright silver phase in each cross-section using automated image-analysis software and calculating the ratio of silver-occupied pixels to total via area. The increase in filling efficiency is partly attributable to the controlled sintering-induced shrinkage, which densifies the silver network without creating new voids, confirming the robustness of our optimized parameters for achieving void-free TGV metallization.

### 3.2. EHD Drop-on-Demand Printing with PWM Control

**Figure 5:**
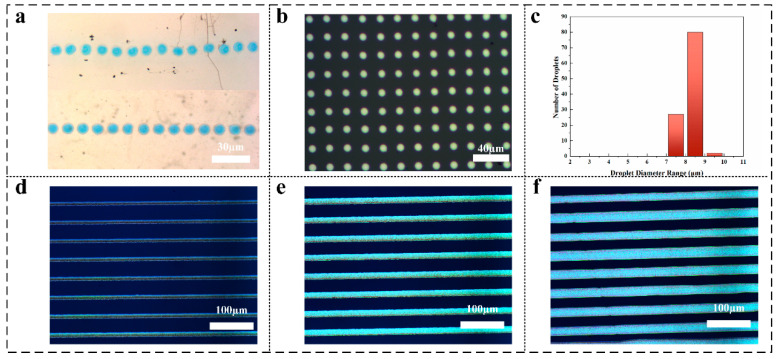
PWM duty cycle effects on EHD Printing: from dot arrays to continuous line width control: (**a**) comparison of unipolar vs. bipolar droplet printing; (**b**) dot array schematic; (**c**) droplet diameter distribution histogram; (**d**) continuous line printed at 80% duty cycle; (**e**) continuous line printed at 85% duty cycle; (**f**) continuous line printed at 95% duty cycle.

With the regime map validated by high-quality, void-free via filling, we now demonstrate in this section how the same EHD platform can directly print fine-pitch circuit patterns on glass. Figure 5a (upper) shows a row of droplets printed under a conventional, unipolar PWM drive: although individual droplets have roughly uniform size, small satellite droplets and tailing appear, and occasional mis–placement arises from electrostatic repulsion between charged droplets and the substrate. In contrast, the lower panel of Figure 5a—using the same duty cycle but alternating polarity every pulse—yields clean, well-centered droplets with negligible satellite formation. Rapid inversion of the electrode polarity actively discharges the substrate after each droplet deposition, thereby restoring a zero-field environment for subsequent droplets and effectively minimizing lateral drift.

Under optimized bipolar PWM at an 85% duty cycle, Figure 5b presents a dot array. Visually, the array is highly regular: droplet diameters appear consistent, and no coalescence bridges are observed. The corresponding size histogram (Figure 5c) confirms this uniformity quantitatively, with a mean diameter of 8.0 ± 0.5 µm, demonstrating sub-10-µm precision at this pitch.

Figure 5d–f show continuous lines printed at three PWM duty cycles—80%, 85% and 95%—on the same substrate. As the duty cycle increases, the average line width grows from approximately 9.1 μm (80%) to 16.7 μm (85%) and up to 21.0 μm (95%). This clear, monotonic relationship demonstrates that simply adjusting the PWM duty cycle provides precise, repeatable control over printed line width, enabling tuning anywhere in the 10–20 μm range to suit specific patterning requirements. Line widths were measured using image-processing software, and the statistical results are presented in Table 1.

### 3.3. Printed Antenna and Performance Validation

**Figure 6:**
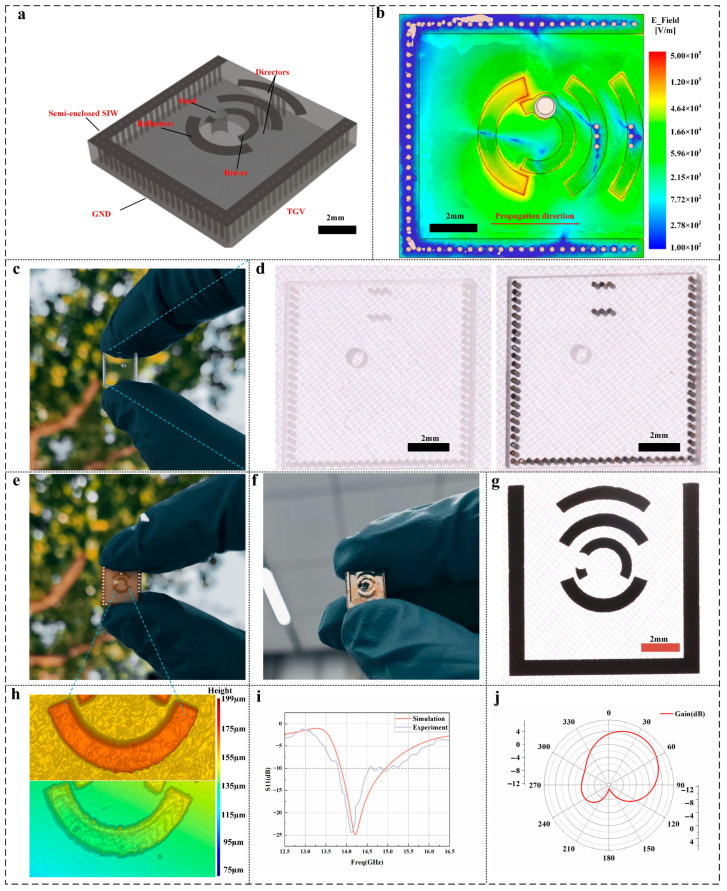
Design, fabrication, and performance of the glass-based SIW antenna. (**a**) Three-dimensional schematic of the semi-enclosed substrate-integrated waveguide (SIW) structure. (**b**) Electric filed intensity of a semi-enclosed SIW antenna (**c**) Photograph of only the TGV array, before any filling or circuit printing. (**d**) Top-view images of the blank glass substrate and the same substrate after nano-silver ink TGV filling (right). (**e**) PCB-fabricated antenna (**f**) EHD-fabricated antenna (**g**) Optical images of the printed circuit patterns. (**h**) Results of profilometer scanning (Top: PCB, Bottom: EHD). (**i**) Measured (blue curve) and simulated (red curve) S_11_ versus frequency. (**j**) Simulated radiation pattern in the azimuthal plane.

Having established high-resolution circuit printing, we next showcase in this section the functional applications of these printed devices. Figure 6a shows the three-dimensional topology of our glass-based SIW antenna. A 10 mm × 10 mm × 1.52 mm borosilicate plate is turned into a semi-enclosed waveguide by plating two parallel rows of TGVs along its edges—these vias emulate perfect electric walls (PEC) and confine the TE_10_ mode within the cavity. A single, centrally located TGV serves as the feed transition: it carries the coplanar feed line from the underside through the glass into the SIW cavity above.

Figure 6b shows the HFSS-simulated electric field magnitude (E) within our semi-enclosed SIW cavity antenna at the design frequency. The metallized via rows and sidewalls impose a PEC boundary, as evidenced by the intense field concentration inside the cavity (up to ~5 × 10^5^ V/m, red/orange regions) and the rapid, evanescent decay of the field outside the SIW walls (green to blue). This exponential drop across the wall confirms that the SIW boundary effectively blocks outgoing waves and reflects electromagnetic energy back into the cavity. As a result, all power is steered toward the open side of the antenna (arrow indicated), enhancing end-fire radiation while suppressing leakage through the closed sides. By integrating feed, reflector, driver and director into a single EHD-printed metal layer on glass, this design achieves a low-profile, high-Q antenna with precise beam control—ideal for transparent or conformal Ku-band applications.

The raw glass substrate immediately after via drilling as depicted in Figure 6c. The borosilicate plate features a dense, regularly spaced array of 200 µm-diameter channels. High-magnification inspection reveals virtually zero debris at the via mouths—critical prerequisites for uniform wetting by the silver ink and for preventing void entrapment during the subsequent EHD filling.

Figure 6d presents side-by-side optical micrographs of the same substrate region before and after EHD filling. In the “before” image (left), each via appears as bright, reflective plugs flush to the surface. After filling, a dark, light-blocking cylinder against the transparent glass background. The antenna topology on a borosilicate plate, where rows of 200 µm × 1.52 mm TGVs form the SIW sidewalls and a central via delivers the feed into the cavity and into an arc-shaped radiating slot above.

Figure 6f shows a photograph of the fully assembled glass-antenna module compared with the PCB-fabricated antenna shown in Figure 6e: the transparent borosilicate plate is held in a precision clamp, and the central through-glass via feed is mated to a standard coaxial connector via a microstrip launch on the underside. The electrodynamically printed SIW walls and arc-shaped slot appear as bright silver features against the clear substrate, highlighting the seamless, mask-free integration of feed, waveguide and radiator in one monolithic element.

A optical micrograph of the EHD-printed SIW sidewalls and radiating slot as Figure 6g shown. The silver conductors maintain a uniform width of 200 µm over their entire length, with razor-sharp edges that show no satellite droplets, edge bulging or pattern distortion. A mirror-like surface finish and consistent grayscale intensity across adjacent lines attest to precise drop-on-demand deposition and perfect coalescence of individual jets. This exceptional feature fidelity directly translates into tight impedance control and minimal geometric deviation from the design. The printing process video can be found in the supporting file S4.

Figure 6h presents a white-light profilometry comparison between a copper trace fabricated by conventional PCB etching and a silver trace produced by EHD-DOD printing. The etched copper exhibits an average surface roughness Sa of 3.34 µm and an average line height of 56.91 µm, with noticeable edge taper and undercut. In contrast, the printed silver trace achieves a Sa of 1.36 µm and a line height of 7.94 µm, featuring nearly vertical sidewalls and excellent planarity. The dramatic reduction in roughness and profile height achieved by EHD-DOD printing minimizes RF scattering and insertion loss in Ku-band operation, while enabling a markedly more compact, low-profile antenna integration.

From the trends shown in Figure 6i, both simulated (red) and measured (blue) reflection coefficients. The simulation predicts a deep S_11_ notch of −26 dB at 14.7 GHz with a −10 dB impedance bandwidth from 13.1 to 15.5 GHz. Experimentally, the resonance shifts to 14.2 GHz with a minimum S_11_ of −18 dB and a measured −10 dB bandwidth of 12.8–16.2 GHz. The ~0.5 GHz downshift and slight bandwidth broadening arise from the finite conductivity and thickness variation of the printed silver traces, as well as minor dimensional tolerances in via diameter and slot width.

Figure 6j shows the measured E-plane radiation pattern, where the main lobe is steered off broadside to a peak at approximately 37°. The half-power beamwidth extends from about 10° to 64°, giving an HPBW of roughly 54° around the 37° peak. The first sidelobes appear at nearly −10 dB below the main beam, and cross-polarization levels remain better than −20 dB across the main lobe. These results confirm that our arc-shaped director in combination with the SIW cavity produces a tilted, high-gain beam exactly as designed—and that the drop-on-demand EHD-printed conductors faithfully realize the intended antenna geometry.

## 4. Conclusions

We have developed a unified, physics-driven EHD printing strategy that simultaneously achieves void-free filling of high-aspect-ratio TGVs and on-demand printing of sub-10 μm conductive traces in a single pass. By constructing a dimensionless regime map—based on steady-state electrostatic simulations, transient level-set modeling, and meniscus-imaging experiments—we identified the operating window for stable cone–jetting and meniscus vibrations. Guided by these insights, we optimized nozzle geometry and ink rheology (selecting silver-nanoparticle suspensions ≥ 2000 cP) and introduced a bipolar, pulse-width-modulated driving waveform that actively neutralizes substrate charge between pulses.

Using this approach, we achieved complete, void-free filling of 200 μm × 1.52 mm TGVs and simultaneous drop-on-demand printing of circuit lines on borosilicate glass. Fabricated substrate-integrated waveguide antennas exhibit an S_11_ minimum of −18 dB at 14.2 GHz, a 12.8–16.2 GHz −10 dB bandwidth, and an 8 dBi peak gain with a 37° beam tilt—results in close agreement with full-wave simulations.

Overall, our integrated regime-mapping framework and bipolar-PWM scheme overcome the traditional trade-offs between ink viscosity, field strength, and charge accumulation, opening a scalable route to high-performance, glass-integrated RF devices and transparent electronics. Although the single-nozzle filling time is 35 s, employing multi-nozzle EHD arrays can dramatically shorten the effective per-via time and improve throughput, making this approach economically promising for RF device manufacturing. Future work will extend this methodology to multilayer 3D interconnects and chemically functional inks for fully printed microelectronic systems.

## Figures and Tables

**Table 1:** Summary statistics of printed line.

Image	Count	Mean (µm)	Min (µm)	Max (µm)
Figure 5d	8	9.1	8.3	10.4
Figure 5e	8	16.7	15.7	17.6
Figure 5f	8	21.0	15.7	24.3

## Data Availability

The data presented in this study are available on request from the corresponding authors.

## Supplementary Materials

The following supporting information can be downloaded at: https://www.mdpi.com/article/10.3390/mi16080907/s1, File S1: Simulation model and ink properties; File S2: Side top-down view of EHD-filled TGV; File S3: Side view of EHD-filled TGV; File S4: EHD priting on glass.
