# Supplementary material for: Integrated Additive Manufacturing of TGV Interconnects and High-Frequency Circuits via Bipolar-Controlled EHD Jetting
Source: Micromachines (Basel). 2025 Aug 2;16(8):907. doi: 10.3390/mi16080907 (PMC12388383; doi:10.3390/mi16080907)
Supplement: Supplementary file 1 [file micromachines-16-00907-s001.zip › S1_Supplementary doc.pdf]

1 Simulation Parameters and Mesh Generation

Table S1. Simulation Material Parameter Settings.

	ρ (kg/m³)	μ (Pa·s)	ε_r	σ_c (S/m)	σ (N/m)	θ (°)
Silver nanoparticle ink	2000	1.20	2.5	1.0	0.06	—
Air	1.225	1.8×10^{-5}	1.0	0	–	—
Borosilicate glass	2500	—	5.0	1×10^{-12}	–	45
Stainless-steel nozzle	8000	—	—	1.4×10^{6}	–	no-slip

Here θ is the static contact angle (glass: 45°; via sidewalls: 80°), and the inlet flow rate for the high-viscosity ink is set to 150 nL/s. This integrated multiphysics framework enables quantitative prediction of field-driven jet dynamics and filling kinetics, ensuring void-free, uniform metallization of through-glass vias.

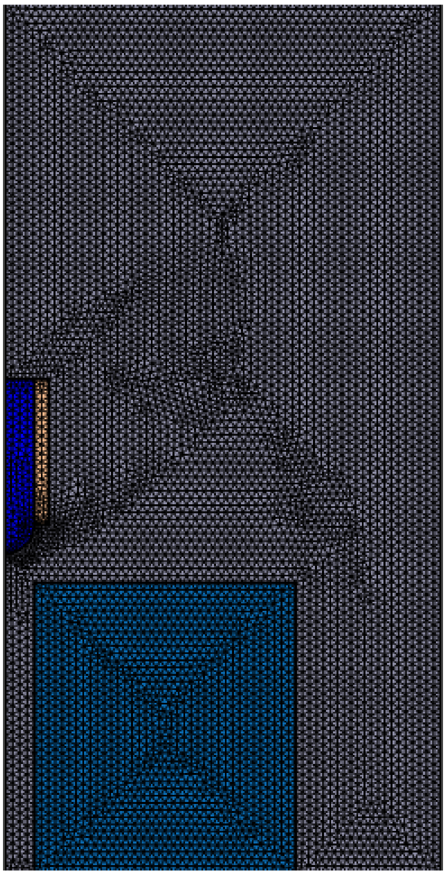

Figure S1. Simulation Mesh Generation.

2 Test Instruments and Ink Parameters

Table S1 Test Instruments

**Equipment**	**Model**	**Key Specifications**
Industrial nano-CT	Phoenix Nanotom® M	• X-ray nanofocus tube: 180 kV/20 W • Smallest effective pixel size (voxel): 0.3 µm • Max. sample Ø: 240 mm; max. height: 250 mm; max. weight: 3 kg • System dimensions: 1 980 × 1 600 × 925 mm; weight ≈ 1 900 kg
FE-SEM	SU8010	• SE resolution: 1.0 nm (15 kV, WD 4 mm) & 1.3 nm (landing voltage 1 kV, WD 1.5 mm) • Beam deceleration down to 100 V landing voltage • Stage travel: X/Y 0–50 mm, R 0–360°, T –5–70°, Z 1.5–30 mm; max. sample Ø 100 mm
3D Laser-scanning confocal microscope	VK-X1000	• Laser wavelength: 404 nm • Vertical resolution: 20 nm; lateral resolution diffraction-limited; scan area up to 50 × 50 mm • Magnification up to 28 800×; field of view 11–7 398 µm
High-voltage power supply	AJ2	• Output voltage: ± 3 kV DC or AC peak• Peak slew rate: > 250 V/µs
Vector network analyzer	R&S® ZNB40	• Frequency range: 9 kHz–43.5 GHz • Dynamic range: up to 140 dB (10 Hz IFBW) • Trace noise: < 0.004 dB RMS (10 kHz IFBW) • Sweep time: 4 ms/401 points; power sweep 98 dB; 2 or 4 ports

Table S2 Parameters of the Ink Used

**Parameter**	**BroadCON-3D750 (High-Viscosity)**	**BroadCON-EHD50 (Low-Viscosity)**
**Ink Type**	Nano-silver conductive paste (for extrusion 3D printing)	Nano-silver conductive ink (for EHD jetting)
**Silver Particle Size**	50 nm	50 nm
**Viscosity**	~2000 cP (adjustable)	12–200 cP (200 rpm @ 25 °C)
**Solid Content**	75–80 wt %	40–80 wt %
**Conductivity / Sheet Res.**	(2–5)×10^{-8} Ω·m	≤ 50 mΩ/□/mil
**Curing Method**	Thermal, IR, 808 nm or 1064 nm laser	130–150 °C for 30 min; NIR or photonic sintering
**Typical Delivery**	Peristaltic pump, screw pump, piston syringe, pneumatic/gear pump, piezo valve	Gravity-fed U-tube (EHD jet)
**Adhesion**	Grade 0 (no peeling)	5B (per ASTM D3359, 3M 810 tape)
**Hardness**	2H	2H
